# Predicting Hospitalization Length in Geriatric Patients Using Artificial Intelligence and Radiomics

**DOI:** 10.3390/bioengineering12040368

**Published:** 2025-03-31

**Authors:** Lorenzo Fantechi, Federico Barbarossa, Sara Cecchini, Lorenzo Zoppi, Giulio Amabili, Mirko Di Rosa, Enrico Paci, Daniela Fornarelli, Anna Rita Bonfigli, Fabrizia Lattanzio, Elvira Maranesi, Roberta Bevilacqua

**Affiliations:** 1 Unit of Nuclear Medicine, IRCCS INRCA, 60127 Ancona, Italy; l.fantechi@inrca.it (L.F.); d.fornarelli@inrca.it (D.F.); 2 Scientific Direction, IRCCS INRCA, 60124 Ancona, Italy; f.barbarossa@inrca.it (F.B.); g.amabili@inrca.it (G.A.); a.bonfigli@inrca.it (A.R.B.); f.lattanzio@inrca.it (F.L.); r.bevilacqua@inrca.it (R.B.); 3 Unit of Radiology, IRCCS INRCA, 60127 Ancona, Italy; s.cecchini@inrca.it (S.C.); l.zoppi@inrca.it (L.Z.); e.paci@inrca.it (E.P.); 4 Unit of Geriatric Pharmacoepidemiology, IRCCS INRCA, 60127 Ancona, Italy; m.dirosa@inrca.it

**Keywords:** radiomics, CT image, hospitalization stay, machine learning, older adults

## Abstract

(1) Background: Predicting hospitalization length for COVID-19 patients is crucial for optimizing resource allocation and patient management. Radiomics, combined with machine learning (ML), offers a promising approach by extracting quantitative imaging features from CT scans. The aim of the present study is to use and adapt machine learning (ML) architectures, exploiting CT radiomics information, and analyze algorithms’ capability to predict hospitalization at the time of patient admission. (2) Methods: The original CT lung images of 168 COVID-19 patients underwent two segmentations, isolating the ground glass area of the lung parenchyma. After an isotropic voxel resampling and wavelet and Laplacian of Gaussian filtering, 92 intensity and texture radiomics features were extracted. Feature reduction was conducted by applying a last absolute shrinkage and selection operator (LASSO) to the radiomic features set. Three ML classification algorithms, linear support vector machine (LSVM), medium neural network (MNN), and ensemble subspace discriminant (ESD), were trained and validated through a 5-fold cross-validation technique. Model performance was assessed using accuracy, sensitivity, specificity, precision, F1-score, and the area under the receiver operating characteristic curve (AUC-ROC). (3) Results: The LSVM classifier achieved the highest predictive performance, with an accuracy of 86.0% and an AUC of 0.93. However, reliable outcomes are also registered when MNN and ESD architecture are used. (4) Conclusions: The study shows that radiomic features can be used to build a machine learning framework for predicting patient hospitalization duration. The findings suggest that radiomics-based ML models can accurately predict COVID-19 hospitalization length.

## 1. Introduction

### 1.1. Overview of COVID-19 and Prognostic Challenges

Severe acute respiratory syndrome coronavirus 2 (SARS-CoV-2), COVID-19, disease has strongly affected global health and still represents a major cause of death worldwide [1]. Along with mortality, the number of infected patients is rapidly and continuously increasing, due to the high contagiousness of the virus [2,3,4]. Nowadays, as of 19 September 2022, there have been more than 609 million confirmed cases of COVID-19 globally [5]. Among severe consequences of this disease, pulmonary, hematological, neurological, and systemic complications may occur, increasing the degree of pathogen lethality [6]. Higher hospitalization and mortality ratio are registered in people aged over 75 years old [6,7]. Because of the severity of this disease, solving issues related to diagnosis, prognosis, and therapeutic path remains a crucial point [8]. In particular, prognostic tools are needed to provide clinicians with insights to optimize treatment strategies, manage the hospitalization of patients both in the wards and intensive care units, and better handle patient follow-up plans [9]. In line with this, X-ray computed tomography (CT) has received clinical and research interest for the management of COVID-9 patients [10,11,12], especially with the development of radiomics, to increase the accuracy of COVID-19 detection [13]. By involving artificial intelligence (AI), in fact, radiomics offers optimistic perspectives through image transformation in mathematical numbers [13], allowing health professionals to retrieve a number of information hidden in medical images and exploit the discriminating power of a large range of features, established with clinical and biological findings [14,15]. It has been demonstrated that radiomics provides a reliable and non-invasive approach to improving efficiency degree in diagnosis [14] and therapy prediction, exploiting different imaging techniques. Indeed, radiomics can be applied to a number of diagnostic imaging techniques. Magnetic resonance imaging radiomics analysis is largely applied in a wide range of disease detection tasks, like the identification of prostate cancer [16], renal carcinoma [17], and brain tumors [18,19]. On the other side, radiomics applied to CT images are increasingly utilized to provide differential diagnosis [20], exploiting AI to make screening predictions of disease progression and the image processing (segmentation) of lung lesion areas. In the case of COVID-19, research studies have been conducted for the detection of the disease through radiomics, including screening patients for other lung infections and identifying the progression of the disease, through machine learning (ML) approaches. For example, Shiri et al. [21] developed prognostic models for the prediction of COVID-19 patients using clinical data along with lung lesion radiomic features extracted from chest CT images, demonstrating that it is possible to effectively predict outcome in COVID-19 patients. Indeed, previous studies have indicated that combining clinical information with radiomics feature analysis has the potential to serve as a substitute for diagnosis, prognosis, and therapy response performance evaluation [15,16,17]. Fu et al. [22] developed an ML architecture able to classify the progression state of COVID-19. In particular, they could discriminate among stable and progressive groups with 80% accuracy. Yip et al. [23] proposed an ML approach to predict disease severity, and their AI model could discretely perform this task (AUC 0.80).

### 1.2. Summary of Existing Research

Table 1 summarizes the current state-of-the-art in the application of ML models for predicting the severity and survival rate for patients affected by COVID19 through radiomics analysis.

Despite these advancements, none of the previous studies have been focused on the prediction of the hospitalization length at the hospital admission through radiomics. Understanding the duration of hospital stay since admission may improve the clinical management and characterization of COVID-19 patients as well as the administration of medical resources by orienting clinical decisions in advance, planning timely the use of medical equipment and interventions and thus developing adapted standards and guidelines for different contexts and low-resources settings.

### 1.3. Research Objective and Hypotheses

The aim of the present study is to build machine learning architectures, exploiting CT radiomics information that is able to predict hospitalization stay of patients affected by COVID-19 from hospital admission. We hypothesize that integrating radiomics with AI-driven predictive models can provide accurate estimations of hospital stay duration, facilitating more effective patient management and resource allocation and creating a positive gap, enhancing the current strategies for predicting hospitalizations.

### 1.4. Structure of the Paper

The paper is structured as follows: Section 2 provides an overview of the dataset and preprocessing techniques used, with the machine learning methodologies employed for predictive modeling. Section 3 presents the experimental results and performance evaluation of the developed models. Section 4 discusses the implications of our findings and potential clinical applications. Finally, Section 5 concludes the paper and outlines future research directions.

## 2. Materials and Methods

**Figure 1:**
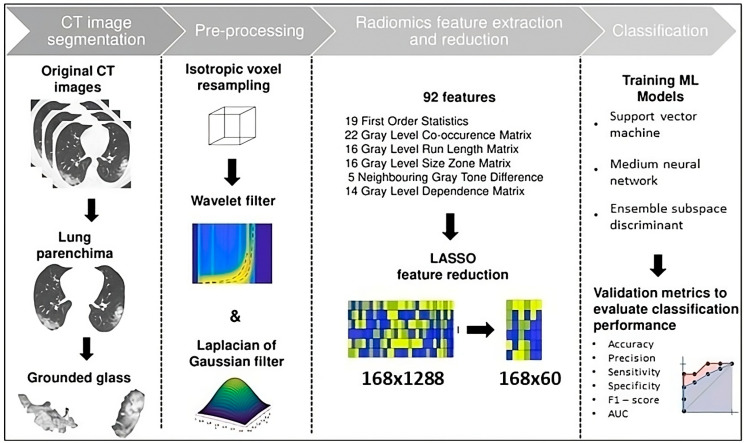
Flowchart of the adopted method protocol.

Figure 1 summarizes all the steps involved in the study design. First, the CT images were segmented from the original CT, thus only the ground glass area was obtained. These sections were resampled and filtered so that they were ready for radiomic feature extraction. Once the radiomics feature dataset was derived, LASSO was applied as a feature reduction technique. Three machine learning algorithms for the length of hospitalization stay prediction were trained and validated from the newly obtained dataset. An evaluation of ML classifier performance based on standard metrics was performed.

### 2.1. Participants

A total of 168 patients hospitalized in IRCCS INRCA for COVID-19 disease, in the period from October 2020 to March 2021, were considered. They were divided by length of hospital stay (LoS, 2 categories): 91 of them were hospitalized for a duration ≤ 14 days while 77 of them for >14 days. The selection of the 14-day threshold for binarizing hospitalization duration in COVID-19 patients is based on clinical and epidemiological evidence. Previous studies have identified similar thresholds, with hospitalization durations typically ranging from 10 to 15 days [24,25] depending on disease severity, patient age, and comorbidities. Clinically, a hospital stay exceeding two weeks often indicates a more severe course, requiring prolonged ventilation or post-acute rehabilitation. The threshold aligns with prior research on COVID-19 outcomes and facilities [26], distinguishing between patients with favorable versus prolonged recoveries. Additionally, preliminary data analysis confirmed a natural division at 14 days, optimizing dataset balance and enhancing the predictive performance of machine learning models.

### 2.2. CT Image Segmentation

As the first evaluation at hospital admission, all the subjects underwent a chest CT scan using a G.E. Bright-Speed ELITE tomograph (General Electric, Milwaukee, WI, USA) at the Radiology Unit of IRCCS INRCA, located in Ancona, Italy. All images were reconstructed with a slice thickness of 1.75 mm and pixel dimensions of 0.5 × 0.5 mm to 0.8 × 0.8 mm. Given the early assessment, the segmentation was aimed at the selection of ground glass areas, which are considered the first and most common alteration in SARS-COV-2 pneumonia. The segmentation consisted of two phases:

- Lung parenchyma extraction: In the first phase, lung parenchyma extraction has been conducted, using Advantage Workstation 4.2 (General Electric, Milwaukee, WI, USA). Images of extracted lung parenchyma and the original acquisitions were then exported in DICOM format and saved for further analysis. In this phase, the images were reviewed by a radiologist and a technician to assess the quality of acquisition and the correct extraction of parenchyma.
- Ground-glass opacity segmentation: The extracted lung images were converted into numerical matrices using Python (ver. 3.8.10), and voxels with Hounsfield unit (HU) values between −760 and −368 in lung parenchyma images were selected to identify GGO regions.

### 2.3. Image Pre-Processing and Feature Extraction

All GGO image matrices were processed with Pyradiomics (ver. 3.0.1). In the pre-processing phase, the images were as follows:

- Resampled to isotropic voxels of one millimeter per size using sitkBSpline for interpolation.
- Filtered through a wavelet filter—8 decompositions, applying all combinations of high- or low-pass filters in each of the three dimensions
- Filtered through Laplacian of Gaussian filter with 5 values of sigma ([1.0, 2.0, 3.0, 4.0, 5.0]).

For each set of images (1 original image and 16 filtered images), 92 features were extracted, as follows: First Order Statistics (19 features), Gray Level Co-occurrence Matrix (22 features), Gray Level Run Length Matrix (16 features), Gray Level Size Zone Matrix (16 features), Neighboring Gray Tone Difference Matrix (5 features), and Gray Level Dependence Matrix (14 features).

To optimize computational efficiency, some high-cost features were excluded. The final dataset contained 1284 features from 168 patients.

### 2.4. Feature Reduction

To perform feature reduction, a last absolute shrinkage and selection operator (LASSO) was used. This technique is widely adopted as a high-dimensional data analysis tool in studies involving radiomics [27,28,29,30,31], since it includes the shrinkage of coefficients, which reduces variance and strongly minimizes bias, and it is able to deal with data that are not linearly related [32]. In general terms, since it is designed to avoid the overfitting of data, LASSO performs better when the number of observations is low, and the number of features is high [32]. In our case, a small number of observations is available (168 patients), and a solid number of features is present (1284 features). The LASSO performs feature selection during model construction by penalizing the magnitude of the regression coefficients [33]. Large regression coefficients are restricted by the imposed penalty factor lambda to reduce overfitting. By increasing the penalty, more regression coefficients shrink to zero, resulting in a more regularized model. LASSO selected the most relevant features, reducing the original dataset from 1284 to 60 features. The loss function reached the minimum value when Lambda was 0.75.

### 2.5. Machine Learning Classification

A number of classical supervised ML algorithms were evaluated using MATLAB 2022a as a programming environment. The entire feature set was used to train algorithms, then a 5-fold cross-validation process was adopted to validate and check the performances of each model. For each of the five folds, a total of 3 metrics was calculated. Accuracy (1), sensitivity (2), specificity (3), precision (4), and F1-score (5) were calculated as:

(1) $$Accuracy=\frac{TP+TN}{TP+FN+TN+FP}$$

(2) $$Sensitivity=\frac{TP}{TP+FN}$$

(3) $$Specificity=\frac{TN}{TN+FP}$$

(4) $$Precision=\frac{TP}{TP+FP}$$

(5) $$F1\text{-}score=2*\;\frac{Precision\;*\;Recall}{Precision+Recall}$$

where *TP*, *TN*, *FP*, and *FN* are true positive, true negative, false positive, and false negative, respectively. The mean of each metrics for all folds represents the overall value of validation metrics. Moreover, the area under receiver operating characteristic (AUC-ROC) curve is calculated to evaluate the ability of the classifier to identify hospitalization days and disease severity. Three classifiers are trained and validated, as follows: (1) linear support vector machine (LSVM), (2) medium neural network (MNN), and (3) ensemble subspace discriminant (ESD). Furthermore, a random search optimization technique has been applied, since it has been demonstrated to be very effective in such types of applications, searching a larger and more promising configuration space [34]. SVM was chosen due to its suitability for both linear and nonlinear binary classification tasks [35]. Further to this point, an extensive use of this algorithm is carried out in many existing studies related to radiomic analysis [36,37,38]. ESD keeps the original data structure by sticking to the original features, which can be helpful for interpretation and providing a direct way for feature ranking [39]. The target describing the duration of hospitalization contains the number of days in which the patient was hospitalized, from the acceptance to the discharge from the hospital. We divided patients into two groups, the first one comprehensive of all the hospitalization duration in the range of 0–14 days and the second one longer than 14 days. In this way, the new target becomes binary, and value 0 is associated for the first class and 1 for the second one. This choice is motivated by the fact that standardized protocols regarding the duration of hospitalization of COVID-19 patients were not available during the study; hence, for this reason, we have identified a threshold that can be relevant from a clinical point of view.

## 3. Results

The demographic and clinical data of the patients are reported in Table 2. The gender division was even, with 104 females and 64 males, and a mean age of participants of 86.5 (±6.4) years. Of the total 168 patients, on the basis of the Clinical Frailty Scale (CFS), 30 patients ranged from very fit to vulnerable individuals; 89 patients ranged from mildly to severely frail, which means they were completely dependent on personal care, and only 44 patients ranged from very severely frail to terminally ill. Three of them were not classified under the CFS scale. A statistically non-significant difference between the two categories related to comorbidity levels is confirmed by *p*-value < 0.05 (0.178). In detail, a specific description of each additional disease to COVID is also given, including infarction, dementia, chronic kidney disease (CKD), hypertension, stroke, chronic obstructive pulmonary disease (COPD), atrial fibrillation, cancer, congestive heart failure (CHF), and diabetes. For each, the number and breakdown between the two categories of LoS is reported.

Statistically significant difference analysis between the two categories for each comorbidity was conducted. The *p*-values confirm that no comorbidities affect LoS except infarction and hypertension, which have a *p*-value of 0.025 and 0.020, respectively. The treatment of each patient is also reported, specifically describing the number of drugs the patient took during the hospital stay, on average 2.7 (±2.0), and whether they received therapy via oxygen. The delivery of oxygen therapy was considered “yes” for those patients whose inspiratory flow of O2 (FIO2) was greater or equal than 21. In this sample, 119 patients received oxygen therapy, and 49 did not. The symptoms exhibited by the patient during hospitalization are also reported, which include cough, dyspnea, diarrhea, vomit, ageusia, and anosmia. The emogas values reported in Table 2 include blood oxygenation status (PO2), with a mean of 64.3 (±13.9), and FIO2, with a mean of 36.7 (±16.4). The associated *p*-value confirms that there is no statistically significant difference among the LoS categories.

The LASSO feature selection process identified 60 key radiomic features, with a strong contribution from GGO-related volume and texture parameters. The importance of filtered features (wavelet and Laplacian of Gaussian transformed images) highlights the relevance of advanced image processing techniques to improve predictive accuracy. From a clinical perspective, these results suggest that early GGO segmentation combined with ML-based radiomic analysis can provide a robust tool for predicting the duration of hospitalization. This information can be integrated into clinical workflows to aid resource allocation, patient monitoring, and early intervention planning.

**Figure 2:**
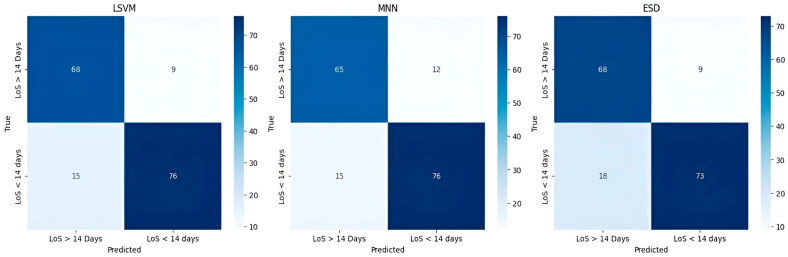
Confusion matrices for the three different predictive models, LSVM, MNN, and ESD.

**Figure 3:**
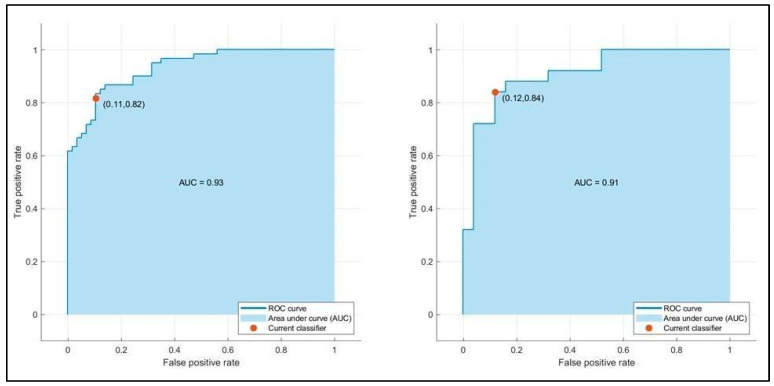
Validation (**left**) and testing (**right**) receiver operating characteristic (ROC)/area under the curve (AUC) for LSVM model in predicting hospitalization days.

**Figure 4:**
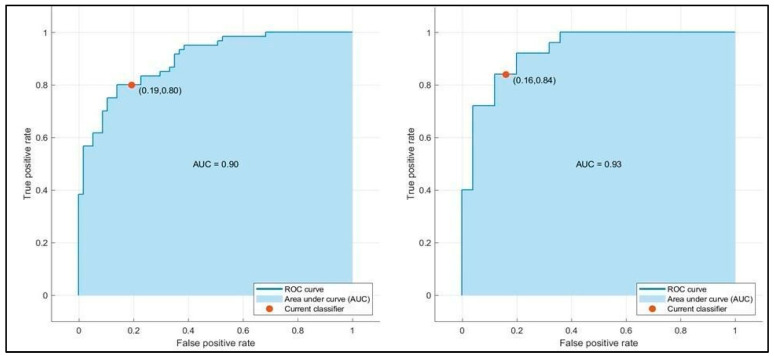
Validation (**left**) and testing (**right**) receiver operating characteristic (ROC)/area under the curve (AUC) for MNN model in predicting hospitalization days.

**Figure 5:**
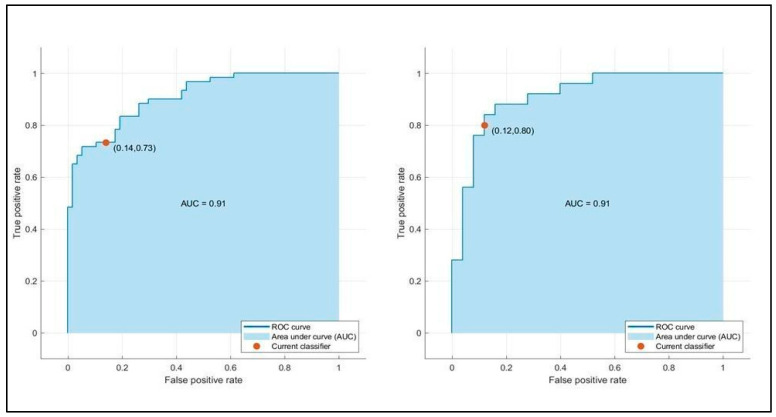
Validation (**left**) and testing (**right**) receiver operating characteristic (ROC)/area under the curve (AUC) for ESD model in predicting hospitalization days.

Table 3 summarizes the training and testing performance of the three ML classifiers. The LSVM achieved the highest predictive performance, with a testing accuracy of 86.0% and an AUC-ROC of 0.93. MNN and ESD also demonstrated reliable predictive capability, with AUC values of 0.93 and 0.91, respectively. The LSVM model outperformed the others in terms of specificity (84.6%) and sensitivity (88.0%), making it the most effective in correctly classifying both short- and long-term hospitalization cases. The MNN model exhibited a slightly lower accuracy (84.0%) but maintained high precision, while the ESD classifier showed good balance but slightly lower performance compared to LSVM. Figure 2 presents the confusion matrices for the three ML models. The LSVM model demonstrated the best classification capabilities, correctly identifying the majority of patients in both hospitalization categories. The false positive rate (patients predicted to have long hospital stays but discharged earlier) was lower in LSVM compared to the other models, enhancing its clinical reliability. Conversely, the MNN and ESD classifiers showed a slightly higher misclassification rate, particularly in predicting long-term hospitalization cases. This suggests that, while these models have strong discriminative power, their ability to predict prolonged hospital stays is slightly less robust than LSVM. Figure 3, Figure 4 and Figure 5 illustrate the ROC curves for each model. The LSVM model achieved an AUC-ROC of 0.93, confirming its superior classification performance. The MNN model, despite achieving a similar AUC, showed minor fluctuations in performance across the validation folds, indicating some degree of model instability. The ESD classifier had a slightly lower AUC of 0.91, suggesting good but less optimal discrimination between short- and long-term hospitalization cases.

## 4. Discussion

At the present, there are a plethora of useful score prediction models to support the early identification of COVID-19 patients that could potentially be affected by the severe progression of the disease. However, the risk related to longer hospitalization still needs to be assessed [40]. In this study a CT radiomics analysis has been performed, involving a group of patients aged 86.5 (±6.4) affected by COVID-19, with the aim of building ML architectures that are able to predict the hospitalization length since the first day of admission of the patients. Previous studies in the field have adopted semi-automatic methods to analyze both or single lungs, by selecting several pulmonary alterations [1,2], such as ground-glass opacities, consolidation, linear opacity, septal thickening and/or reticulation, crazy-paving pattern, air bronchogram, pleural thickening, halo sign bronchiectasis, nodules bronchial, wall thickening, or reversed halo sign. However, no other study was focused only on CT ground-glass opacities (GGO), which appears to be the first and most common alteration (>70%) in early stages of COVID-19, involving bilateral ground-glass opacities, mainly peripheral, with or without consolidation or visible intralobular lines [3]. In addition, the atypical initial imaging presentation of consolidative opacities superimposed on GGO may be found mainly in the older population. Multiple lobe involvement, in fact, was more common in older people than in other age groups, suggesting the high relevance of this parameter in the case of elderly patients [41]. In the present study, a prediction model of hospitalization length based on data extracted only from GGO was developed, with the aim of providing a faster and reliable strategy to detect patients early that may require a higher degree of medical resources. As the elderly represents a high-risk cluster for the rapid clinical deterioration in cases of COVID-19, due to immunosenescence, multimorbidity, and polypharmacotherapy, strategies for early individualized therapeutic interventions are imperative [42]. For this purpose, the three ML classifiers have confirmed the hypothesis that radiomics data from GGO contains enough information about disease evolution, allowing the discrimination between “long-term hospitalization” (>14 days) and “short/medium-term hospitalization” (≤14 days). Among them, LSVM shows the best performance in terms of accuracy. In this study, the degree of radiological severity, defined as the volume of the lung’s involvement, shows its own importance in discriminating the length of hospitalization, as evidenced by the LASSO selection of the features related to GGO volumes. Moreover, a great contribution was given by features extracted from images where Laplacian of Gaussian and wavelet filters were applied. This result suggests that extracted data from GGO, together with the radiomic features that underpin the quality of alterations in such areas, may allow for the early identification of the disease’s evolution towards worsening (i.e., consolidation, fibrosis) or improvement. In line with this, it can be said that GGO may be considered as an intermediate radiological feature that has the potential to evolve towards other alterations or regress to “restitutio ad integrum”. The use of filters applied to GGO was intended to provide a comprehensive assessment, despite their potential to introduce noise or blurring artifacts. However, given the methodological framework applied post feature extraction, LASSO was employed to select the most informative features from a mathematical and statistical perspective. This approach allowed for opportunistic and posteriori reasoning, wherein features were extracted regardless of their initial suitability for GGO characterization—whether due to the intrinsic nature of the filter or the broad range of kernel values utilized. Nevertheless, the subsequent application of LASSO, serving as a dimensionality reduction technique and widely recognized in radiomic analysis, ensured the retention of the most relevant features. Indeed, most of the features outcoming LASSO reduction come from filtered images. This information seems to highlight that filters improve quality or at least quantity of correlated features taken from the image, confirming the usefulness of filtering [5]. Among them, features related to GGO volumes are relevant to predict the length of the hospitalization. The mask, in fact, has been performed on GGO, representing its volume and the overall volume of the ground glass areas, considered as a CT index of disease severity. Despite the relevance of this study, there are several limitations to mention, for example the small size of the sample included. Moreover, the inclusion of information on clinical history and pharmacological therapies should be considered in the future, together with inclusion of respiratory indices for improving the prognostic capability and accuracy of the model. However, the study represents a relevant attempt for the application of CT radiomics for robust prognostic modeling, as the algorithm was effective in identifying the hospitalization stay coherently with the clinical profile of the patients. This result highlights the undeniable wealth of radiomics to support health professionals and hospital management teams to a better understand diseases and the identification of effective treatment options, by combining artificial intelligence with clinical assessment for the prediction of health outcomes [42].

## 5. Conclusions

The study underscores the importance of leveraging CT radiomics analysis in predicting length of hospitalization stay for COVID-19 patients. By focusing solely on ground-glass opacities (GGO) and employing machine learning algorithms, the research aimed to provide a rapid and reliable strategy for identifying patients requiring extensive medical resources, particularly in the context of an aging population vulnerable to severe disease progression. The findings suggest that radiomics data from GGO contains valuable information for discriminating between long-term and short/medium-term hospitalization, with LSVM exhibiting superior performance in terms of accuracy.

Our results support the hypothesis that by integrating radiomics with machine learning, the duration of hospitalization can be estimated. The ability to classify patients into short-term (≤14 days) and long-term (>14 days) hospitalization groups from the time of admission is a valuable contribution to clinical decision making, allowing for early intervention and improved treatment strategies.

The main advantage of the proposed methodology is its ability to provide early predictions based on imaging data, reducing the need for more extensive clinical or laboratory assessments. However, the sample size and the lack of integration with clinical and laboratory data appear to be limitations for the present research.

Future studies should focus on expanding the dataset to include more diverse patient populations and integrating additional clinical and biological markers to refine prediction accuracy. Furthermore, the application of deep learning techniques and advanced feature selection methods could further improve the robustness of predictions based on radiomics techniques.

## Figures and Tables

**Table 1:** Systematic overview of existing research.

Article	Shiri et al. [21]	Fu et al. [22]	Yip et al. [23]
Aim	Develop prognostic models for survival prediction in COVID-19 patients using clinical, radiomic data from chest CT	Investigating the performance and robustness of radiomics in predicting the severity of COVID-19	Use radiomic signatures derived from whole-lung machine learning to assess the prognosis of patients with COVID-19
Study design	Retrospective	Retrospective	Retrospective
Patients (n)	152	1110	64
Data	Clinical data, radiological scores, radiomic features from lungs and segmented lesions on CT scan	Chest CT images and severity classifications (mild, moderate, severe)	Clinical data and lung CT scans
Segmentation	Whole lung and infective lesion manually segmented	Whole lung segmented through watershed algorithm	Whole lung segmented through automatic AI software
Feature extraction	130 features (first order, shape, GLCM, GLRLM, GLSZM, NGTDM, GLDM)	107 unfiltered features (first order, shape, GLCM, GLRLM, GLSZM, NGTDM, GLDM)	Shape, GLCM, RLM, GLZSM)
ML Models	XGBoost	Logistic regression	Support vector machine
Results	Combined model (lung + lesion + clinical data) showed the best prognostic performance (AUC = 0.95, accuracy = 0.88, sensitivity = 0.88, specificity = 0.89)	For mild vs severe classification, AUCvalid ≈ 0.80, moderate vs severe prediction was less accurate (AUC ≈ 0.65)	For classifying between stable and progressive infection with AUC = 0.833, sensitivity = 80.95%, specificity = 74.42%

**Table 2:** Sample characteristics according to length of stay.

	Total	LoS ≤ 14 days	LoS > 14 days	*p*
	n = 168	n = 91	n = 77	
Female gender, n (%)	104 (61.9%)	64 (70.3%)	40 (51.9%)	**0.015**
Age, mean ± sd	86.5 ± 6.4	86.3 ± 6.9	86.7 ± 5.7	0.727
CFS categories, n (%)				0.178
0–3	30 (17.9%)	14 (15.4%)	16 (20.8%)	
4–7	89 (53.0%)	48 (52.7%)	41 (53.2%)	
8–9	44 (26.2%)	24 (26.4%)	20 (26.0%)	
NA	3 (2.9%)	5 (5.5%)	0 (0.0%)	
**Comorbidities**				
Infarction, n (%)	15 (8.9%)	4 (4.4%)	11 (14.3%)	**0.025**
Dementia, n (%)	57 (33.9%)	32 (35.2%)	25 (32.5%)	0.713
CKD, n (%)	39 (23.2%)	21 (23.1%)	18 (23.4%)	0.963
Hypertension, n (%)	111 (66.1%)	53 (58.2%)	58 (75.3%)	**0.020**
Stroke, n (%)	20 (11.9%)	7 (7.7%)	13 (16.9%)	0.067
COPD, n (%)	21 (12.5%)	13 (14.3%)	8 (10.4%)	0.447
Atrial fibrillation, n (%)	49 (29.2%)	25 (27.5%)	24 (31.2%)	0.599
Cancer, n (%)	37 (22%)	16 (17.6%)	21 (27.3%)	0.131
CHF, n (%)	41 (24.4%)	20 (22%)	21 (27.3%)	0.426
Diabetes, n (%)	39 (23.2%)	20 (22%)	19 (24.7%)	0.680
**Treatments**				
Number of drugs, mean ± sd	2.7 ± 2.0	2.0 ± 1.4	3.4 ± 2.3	**<0.001**
Oxygen therapy, n (%)	119 (70.8%)	62 (68.1%)	57 (74.0%)	**0.028**
**Symptoms**				
Cough, n (%)	38 (22.6%)	17 (18.7%)	21 (27.3%)	0.235
Dyspnea, n (%)	91 (54.2%)	54 (59.3%)	37 (48.1%)	0.070
Diarrhea, n (%)	14 (8.3%)	6 (6.6%)	8 (10.4%)	0.420
Nausea, n (%)	4 (2.4%)	2 (2.2%)	2 (2.6%)	0.899
Vomit, n (%)	12 (7.1%)	7 (7.7%)	5 (6.5%)	0.707
Conjunctivitis, n (%)	2 (1.2%)	1 (1.1%)	1 (1.3%)	0.929
Ageusia, n (%)	1 (0.6%)	0 (0%)	1 (1.3%)	0.286
Anosmia, n (%)	1(0.6%)	0(0%)	1(1.3%)	0.286
**Emogas Analysis**				
PO2, mean ± sd	64.3 ± 13.9	63.8 ± 11.2	64.9 ± 16.4	0.621
FIO2, mean ± sd	36.7 ± 16.4	34.4 ± 15.5	39.1 ± 17.1	0.094

LoS = length of hospital stay; n = number of subjects; sd = standard deviation; CFS = Clinical Frailty Scale; CKD = chronic kidney disease; COPD = chronic obstructive pulmonary disease; CHF= congestive heart failure; PO2 = partial oxygen pressure [mmHg]; FIO2 = inspiratory oxygen flow; NA = not available.

**Table 3:** Training and testing metrics describing performance of three classifiers.

	LSVM Train/Test	MNN Train/Test	ESD Train/Test
Accuracy (%)	85.4/86.0	80.3/84.0	79.5/84.0
Precision (%)	82.2/84.6	79.3/84.0	75.4/81.5
Sensitivity (%)	89.5/88.0	80.7/84.0	86.0/88.0
Specificity (%)	81.784.0	80.0/84.0	73.3/80.1
F1-score (%)	85.7/86.2	80.0/84.0	80.3/84.6
AUC	0.91/0.93	0.90/0.93	0.91/0.91

LSVM = linear support vector machine, MNN = medium neural network, ESD = ensemble subspace discriminant.

## Data Availability

The datasets generated, used, and analyzed during the trial and its preceding pilot trial are or will be available from the corresponding author upon reasonable request.

## Abbreviations

The following abbreviations are used in this manuscript:

AI	Artificial Intelligence
AUC-ROC	Area Under Receiver Operating Characteristic
CFS	Clinical Frailty Scale
CHF	Congestive Heart Failure
CKD	Chronic Kidney Disease
COPD	Chronic Obstructive Pulmonary Disease
CT	Computed Tomography
ESD	Ensemble Subspace Discriminant
FIO2	Inspiratory oxygen flow
FN	False Negative
FP	False Positive
GGO	Ground-Glass Opacities
LASSO	Last Absolute Shrinkage and Selection Operator
LoS	Length of Hospital Stay
LSVM	Linear Support Vector Machine
ML	Machine Learning
MNN	Medium Neural Network
PO2	Partial Oxygen Pressure
TN	True Negative
TP	True Positive
